# Temporal changes in the potential geographic distribution of *Histiotus velatus* (Chiroptera, Vespertilionidae), the “decade effect”

**DOI:** 10.1002/ece3.8333

**Published:** 2021-12-01

**Authors:** Liriann Chrisley Da Silva, Rafaela Gonçalves Almeida, Pablo Henrique da Silva, Monik Oprea, Poliana Mendes, Daniel Brito, Thiago Bernardi Vieira

**Affiliations:** ^1^ Programa de Pós‐Graduação em Biodiversidade e Conservação Faculdade de Ciências Biológicas Universidade Federal do Pará Altamira Brazil; ^2^ Laboratório de Ecologia Aplicada e Conservação Departamento de Ecologia Instituto de Ciências Biológicas Universidade Federal de Goiás Goiânia Brazil; ^3^ Programa de Pós‐Graduação em Recursos Naturais do Cerrado – RENAC Universidade Estadual de Goiás Anápolis Brazil; ^4^ Theoretical Metacommunity and Landscape Ecology Laboratory Departamento de Ecologia Instituto de Ciências Biológicas Universidade Federal de Goiás Goiânia Brazil; ^5^ Programa de Pós‐Graduação em Genética e Biologia Molecular Departamento de Genética Instituto de Ciências Biológicas Universidade Federal de Goiás Goiânia Brazil; ^6^ Département de Phytologie Faculté des sciences de l’agriculture et de l’alimentation Université Laval Québec QC Canada; ^7^ Programa de Pós‐Graduação em Ecologia e Evolução Departamento de Ecologia Instituto de Ciências Biológicas Universidade Federal de Goiás Goiânia Brazil

**Keywords:** conservation, ecological niche modeling, research center, species distribution models, species occurrence, Wallacean Deficit

## Abstract

We investigated how the potential distribution of *Histiotus velatus* is affected by the addition of new records over decades (decade effect). Assuming that (1: hypothesis of the effect of the decade) the addition of new occurrence records over time increases the potential size of the species distribution; and (2: Wallacean distance hypothesis) over the years, the new points added are increasingly distant from the research centers. Considering the geographic knowledge gap of this species, our objective is to report a new record of this species and estimate its potential distribution in South America through environment niche models (ENMs). For this, we compiled records of occurrence of species, selected from 1900 to 2015. We used 19 bioclimatic variables available in the WorldClim database to estimate the potential distribution of the species, and we used three modeling algorithms: Maximum Entropy (MXT), Random Forest (RDF), and Support Vector Machine. To test the Wallacean distance hypothesis, we calculated the Euclidian distance from occurrences to bat research centers in Brazil, located using a national researchers’ information dataset (“Plataforma Lattes”). To test the hypothesis of the decade effect, we used the beta regression analysis, taking conservative and non‐conservative approaches. The results showed that the predicted area expanded and retracted with the addition of new occurrences over the decades, with an improvement in the accuracy of models. Most records are located in the southeastern region of Brazil, but algorithms predicted areas in regions where there are no records. Only the conservative approach has had a positive relationship over the decades. The distance from new points does not increase over the years of research centers.

## Graphical abstract

We investigate how the potential distribution of this species changes with the addition of new records over the decades (decade effect). Assuming that (1: hypothesis of the effect of the decade) the addition of new occurrence records over time increases the potential size of the species distribution; and (2: Wallacean distance hypothesis) over the years, the new points added are increasingly distant from the research centers. Considering the geographic knowledge gap of *Histiotu velatus*, our objective is to report a new record of this species and estimate its potential distribution in South America through ENMs.

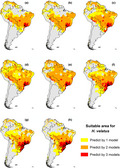

## 1 INTRODUCTION

Scarce geographical data about the distribution of species are often associated with insufficient or inefficient sampling effort throughout time (the Wallacean shortfall; Hortal et al., 2008; Lomolino, 2004). This gap can be minimized by prioritizing sampling in areas with low sampling effort, but this is not always a simple task. For instance, the Wallacean shortfall should be smaller and decrease faster for species originally distributed near large research centers, roads, and easily accessible sites than for those distributed in isolated areas (Hortal et al., 2007; Lobo, 2008; Romo et al., 2006). This is aggravated by the resource shortages for biodiversity inventories that impede the scientific community to find more profitable sampling areas (Hortal et al., 2008; Reddy & Dávalos, 2003). Additionally, there can be temporal biases due to historical contingencies, since data collected in a non‐systematic way can limit the reliability of the species distribution, resulting in an incomplete description of its niche. However, it is possible to use statistical tools to minimize this problem and identify possible geographical and temporal knowledge gaps in species distribution.

Ecological Niche Modeling (ENM) is a statistical procedure often used to identify suitable sites for species occurrence (Peterson & Soberón, 2012), providing essential data for planning biodiversity inventories and conservation actions (Franklin, 2013). This method creates environmental response curves from the species’ known distribution and then estimates area suitability based on the environmental conditions of those locations (Austin et al., 1990). As the number of unique occurrences increases, the models’ predictions become more precise, because ENMs’ accuracy often depends on the amount of single information about the species geographical distribution (Hernandez et al., 2006; Stockwell & Peterson, 2002) and may, therefore, reduce the Wallacean shortfall. Analyzing the increase in information through time might help to understand the spatial and temporal bias on species’ geographic distribution (Hortal et al., 2007). Countries in the tropics, such as Brazil, hold the greatest biodiversity on the planet; however, the real knowledge of the distribution of many species is skewed (Collen et al., 2008; Kier et al., 2005; Santos et al., 2011). Due to its intense spatial variation, difficult access in some regions, the accelerated devastation of ecosystems and the lack of resources for sampling on field (De Marco & Vianna, 2005; Grand et al., 2007; Yang et al., 2013). That of real knowledge with historically neglected data affects the performance of ENMs in this task (Hortal et al., 2007).

Bat species are one of the groups that still face a major Wallacean gap in Brazil. For example, only 10% of the Brazilian territory has been sampled over time and almost 60% of the country does not have a single species occurrence record (Bernard et al., 2011). No biome is considered to be well sampled, and the regions of the Brazilian Amazon, Caatinga and Pantanal are undersampled (Bernard et al., 2011; Bernard & Sampaio, 2008). The South and Southeast regions of Brazil have a higher density of records, possibly justified by the greater concentration of bat research centers, easy logistics of sampled areas and less sampling effort in relation to the others (Brito et al., 2009; Hortal et al., 2008, 2007). However, even the widely distributed species show sample bias due to the low capture rate (Voss & Emmons, 1996).

In addition to these problems, the methods used to sample bat individuals may restrict the number of species captured, leading to an incomplete occurrence record (MacSwiney et al., 2008). Aerial insectivores’ bats, such as *Histiotus velatus* (Chiroptera, Vespertilionidae), are known to fly above the forest canopy (Berry et al., 2004) and are hardly caught in mist nets, the most common bat sampling method. This fact possibly may intensify the lack of information on the geographical distribution of this species. Although it is an insectivorous bat registered in natural, semi‐urban, and urban areas, well adapted to the habitat modifications (Bernardi et al., 2009; Talamoni et al., 2014; Tavares et al., 2010), and widely distributed throughout South America (including Bolivia, Paraguay, Argentina, Peru, and Brazil; Gardner, 2008), it was classified as data deficient (González & Barquez, 2016; Leibold et al., 2004) due to the lack of recent information about its extent of occurrence, status, and ecological requirements (e.g., Arumoogum et al., 2019; Scherrer et al., 2019; Schoeman et al., 2015).

Solving the Wallacean gap is, therefore, an important task for the scientific community (Hortal et al., 2015), and the use of well‐established technologies and protocols can help to increase the effectiveness of sampling efforts (Hortal et al., 2015). On the contrary, assuming the existence of a temporal bias to understanding how collection efforts have been distributed in space may improve the targeting for new samplings. Considering the geographical knowledge gap and possible sampling biases in *H*. *velatus*, our goal is to report a new record of that species in the Goiás state and estimate its potential distribution in South America using ENMs. Additionally, we investigate how this species’ potential distribution changes with the addition of new records over the decades, which we call “decade effect.” Ultimately, we hypothesize that (1: decade effect hypothesis) the addition of new occurrence records over time increases the potential distribution size of the species; and (2: Wallacean distance hypothesis) over the years, the newly added points are further away from research centers.

## 2 METHODS

### 2.1 Species distribution database and data treatment

We compiled occurrence records of *H*. *velatus* available from SpeciesLink (http://www.splink.org.br/index?lang=pt) and GBIF (https://www.gbif.org/). We supplemented our geographical distribution database with records available in scientific articles using the following search code in the Web of Science platform: “bat*” OR “species list” OR “*Histiotus velatus*” OR “*H*. *velatus*”. We selected only the occurrence records since 1900 because the original data were incompatible with the range of the environmental dataset. Furthermore, we excluded the following records: (1) undated occurrence records; (2) records without coordinates; and (3) outside the Neotropical region. Therefore, to investigate the effect of new occurrences over the years, we split the data into eight portions: (1) 1900 to 1950; (2) 1900 to 1960; (3) 1900 to 1970; (4) 1900 to 1980; (5) 1900 to 1990; (6) 1900 to 2000; (7) 1900 to 2010; and (8) 1900 to 2020 with the addition of the new occurrence record localized in the city of Goiânia, Brazil.

### 2.2 Environmental variables and Ecological Niche Models (ENMs)

We used 19 bioclimatic variables (resolution of 9.4 × 9.4 km) for the entire Neotropical realm, available in the WorldClim database (http://www.worldclim.org/). These variables are derived from monthly temperature and precipitation values sampled throughout 1960–1990. Also, these data are often used in ecological modeling techniques to estimate the potential distribution of species (e.g., Lee et al., 2012; Lisón & Calvo, 2013; Sattler et al., 2007). To reduce multicollinearity in our dataset, we performed a Principal Component Analysis (Legendre & Legendre, 2012) and used the eigenvalues as environmental variables. Then, we selected only the axes that represent an explanation equal to or greater than 95% (De Marco and Nóbrega, 2018), using these axes as model variables.

We fit models using three algorithms: Maximum Entropy (MXT; Phillips et al., 2004, 2017), Random Forest (RDF; Prasad et al., 2006), and Support Vector Machine (SVM; Guo et al., 2005). RDF and SVM algorithms require species’ absence data, but these data were not found for *H*. *velatus* in the literature. Therefore, we created 50 pseudo‐absences based on an environmental envelope to allocate pseudo‐absences only in places considered unsuitable for the occurrence of *H. velauts* (Engler et al., 2004). In the case of MXT, models are fitted by differentiating between occurrence records and a 10,000 background points randomly sampled throughout the study area.

We evaluated ENMs using a geographical partition (Muscarella et al., 2014; Roberts et al., 2017). We divided the study area as a checkerboard, which splits the occurrence data into two datasets, and selected each dataset alternately to fit and evaluate. This step allows to evaluate model predictive capacity, as the geographical partition reduces the spatial correlation between datasets used to fit and evaluate the models. Then, we measure model predictive capacity by its value for true skill statistics (TSS), true‐positive rate, and true‐negative rate. This procedure is considered appropriate in studies on geographic distributions of species (Allouche et al., 2006).

We converted the suitability models into presence and absence maps using a threshold at which the sum of the sensitivity and specificity is highest (Allouche et al., 2006). Then, we produced assembled maps using the sum of the binary maps derived from the three algorithms. We used the ENMTML package (Andrade et al., 2020; https://github.com/andrefaa/ENM_TheMetaLand) in R environment (R Core Team, 2021) for all modeling procedures.

### 2.3 Research center data

Brazil is the second country with the highest bat richness; however, all of its biomes have a lack of information on the occurrence of species distribution (Bernard et al., 2011). We selected the main research centers that are developing or have developed surveys about bats in Brazil. For this, we conducted a search by topic in the Lattes platform (http://lattes.cnpq.br/) using the keyword “Quiroptera” (in Portuguese). We chose only those researchers that fall in one of the CNPq's Productivity Researchers categories: 1A, 1B, 1C, 1D, and 2. Furthermore, we established as criteria: (1) research projects about bats; (2) published articles about bats; and (3) academic guidance in bats studies. Included researchers present at least two of these three criteria. In situations in which researchers participated in more than one research center during their career, we choose the location where those professionals spent more time working with bats. We used the Google Earth Pro software to consult the geographic coordinates of the research centers.

### 2.4 Statistical analyses

To test the decade effect hypothesis, we performed beta regression analysis (Ferrari & Cribari‐Neto, 2004) between the number of records over the decades and the proportion of predicted areas, assuming conservatism and non‐conservatism approaches. The conservatism approach considers only the areas predicted by all three algorithms, whereas the non‐conservatism approach considers all the areas predicted by any algorithm. We chose the beta regression analysis because our response variable is restricted to a range of 0 to 1. We performed this analysis in the *betareg* package in software R (Cribari‐Neto & Zeileis, 2010).

For the Wallacean distance hypothesis, we calculated the Euclidean distance between each occurrence record to the closest research center using the *raster* package in the R software (Hijmans et al., 2020). In addition, to reduce a possible forced relationship caused by the excessive number of records, we performed a weighted linear regression considering the total distances calculated for each year as the weight. Then, we related the maximum distance obtained per unit of time to its respective year. We used the highest values observed per year to find out if further away areas from research centers are sampled over time. We also performed the analysis in the R software, using the lm function of the stats package (R Core Team, 2020).

## 3 RESULTS

### 3.1 New record of the species *H. velatus*

We collected the new record of *H*. *velatus* near a pond on the Agronomy School at Federal University of Goiás—Campus II in the municipality of Goiânia, GO (Long −49.28155556 W; Lat −16.62708333 S). This individual is an adult male with abdominal testicles sampled in a mist net. We used the large ears of the species to differentiate *H*. *velatus* from other species of the same genus. The ear's tips of the *H*. *velatus* are beyond the snout, the width of the median lobe of the ear being at least three times the total length of the ear (Gardner, 2008). We collected the following external measurements of the specimen: forearm length = 46.6 mm; body length = 115 mm; tail length = 54 mm; foot length = 6 mm; length ear = 31 mm; tragus length = 14 mm; and weight = 10 g. We deposited the specimen in the Zoological Collection of the Federal University of Goiás under the number ZUFG 110.

### 3.2 Testing the decade effect hypothesis

**FIGURE 1:**
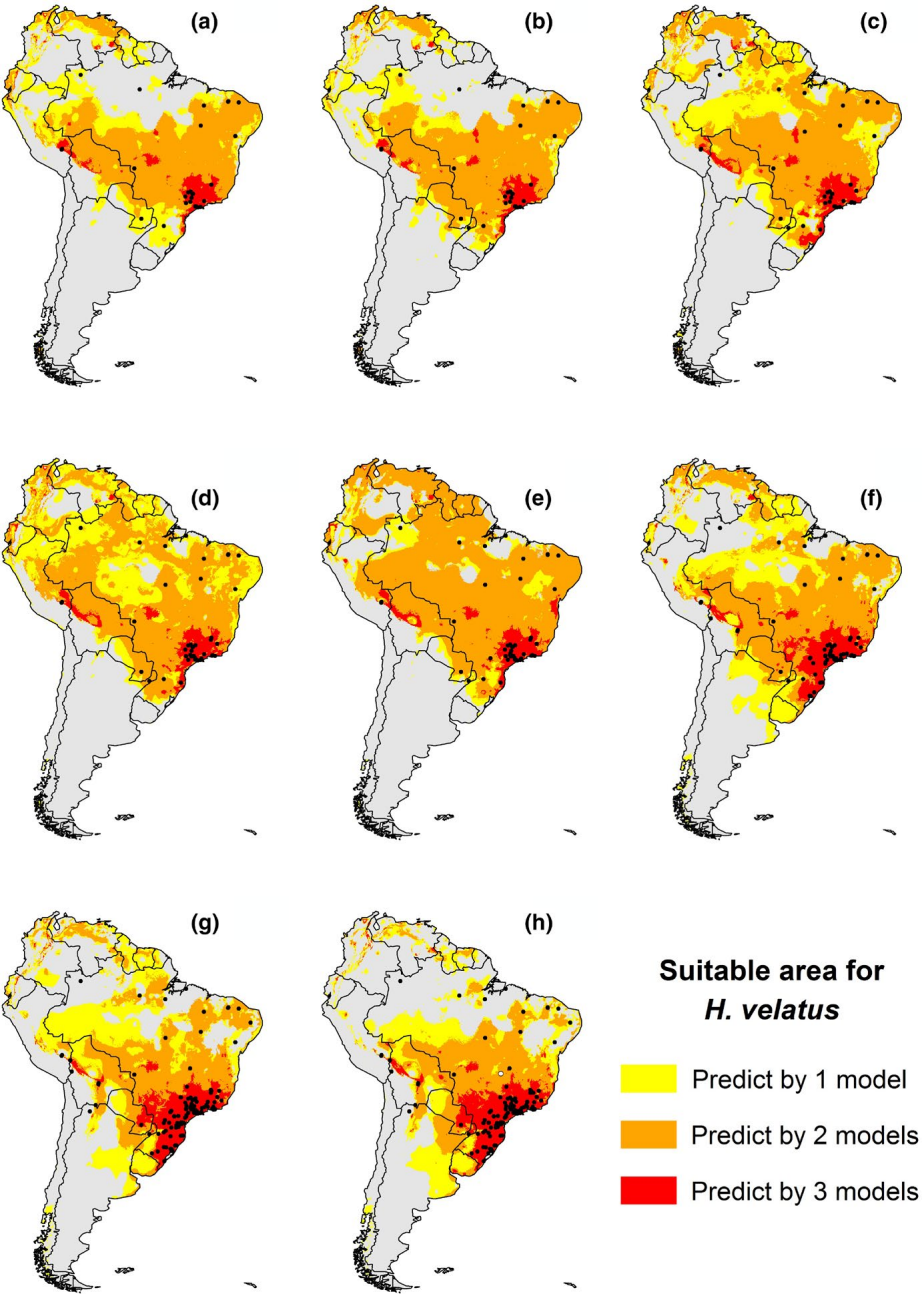
Ecological Niche Models to investigate the effect of the new added points in the potential distribution of *H*. *velatus* at different periods (what we call a “decade effect”). The dataset was subdivided into eight portions: (a) 1900–1950; (b) 1900–1960; (c) 1900–1970; (d) 1900–1980; (e) 1900–1990; (f) 1900–2000; (g) 1900–2010; and (h) 1900–2020. The colors represent the number of algorithms that agreed to predict the potential areas for this species occurrence. The potential distribution in yellow means that only one algorithm predict this areas, in orange are two algorithms, and in red are three algorithms. Areas predicted as unsuitable have gray color. The points represent the occurrence records used in each period, being that the black points were obtained from literature and the white point is a new record

We found 153 occurrence records after data cleaning, with the highest number of new registers in the period 2000–2010 (Table 1). The majority of the records used in the models are located in the southeastern region of Brazil. In addition, we observed that the predicted area has expanded and retracted over the decades (Figure 1). Also, it is possible to observe an improvement in the accuracy of the models with the addition of new data. Algorithms’ performances over the decades varied considerably, evidencing reasonable (close to 0.5) and good evaluations (close to 0.7). Furthermore, in the last two decades, all evaluations were higher than 0.7. Overall, there is a consensus among the predictions of the algorithms for the southeastern Brazil. In addition, these algorithms predicted suitable areas in countries where there were no records of *H*. *velatus*, such as French Guiana, Suriname, Guiana, Uruguay, Venezuela, Colombia, and Ecuador.

**TABLE 1:** Distribution of the 153 occurrence points of *H*. *velatus* in the South America according to the timespan among the decades

Period	Added points	Total points	TSS		
			MXT	RDF	SVM
1900–1950	30	30	0.536	0.790	0.608
1900–1960	1	31	0.596	0.620	0.582
1900–1970	9	40	0.485	0.759	0.703
1900–1980	8	48	0.589	0.860	0.750
1900–1990	3	51	0.708	0.896	0.792
1900–2000	23	74	0.637	0.598	0.831
1900–2010	70	144	0.781	0.812	0.861
1900–2020	9	153	0.745	0.752	0.821

Note

Also, we present the values of TSS as a measure of performance evaluation of the models.

The percentage variation of the proportion of predicted areas highly suitable considering all the presented algorithms was low, varying from 0.3 to 0.8, increasing on average. However, when we perform the analysis for the predicted area by conservatism approach, we find a positive relation. Only the conservatism approach had a positive relationship between the appropriate areas foreseen and the increase in new records over the decades. The proportion of the predict area by non‐conservatism approach do not have relation with the addition of new points over the decades (β = 0.002; *p* = .31).

### 3.3 Testing the Wallacean distance hypothesis

**FIGURE 2:**
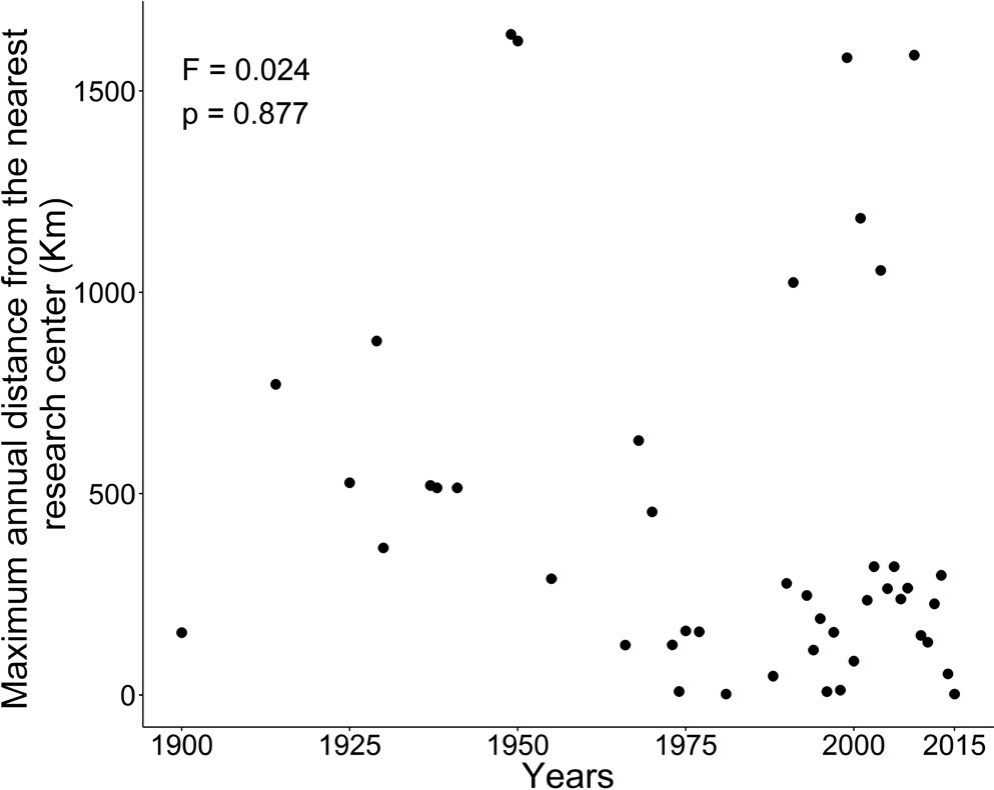
Hypothesis of the Wallacean distance, using Euclidean distance between each occurrence record to the nearest research center and relating the maximum distance obtained per unit of time to its respective year to test the addition of new research points over the years

When we test the Wallacean distance hypothesis, we found that the distance of the new added points to nearest research centers does not increase over the years (*R* ^2^ = −.022, *F* = 0.024, *p* = .877; Figure 2). Thus, possibly the samplings remain spatially biased even after a century of studies.

## 4 DISCUSSION

**FIGURE 3:**
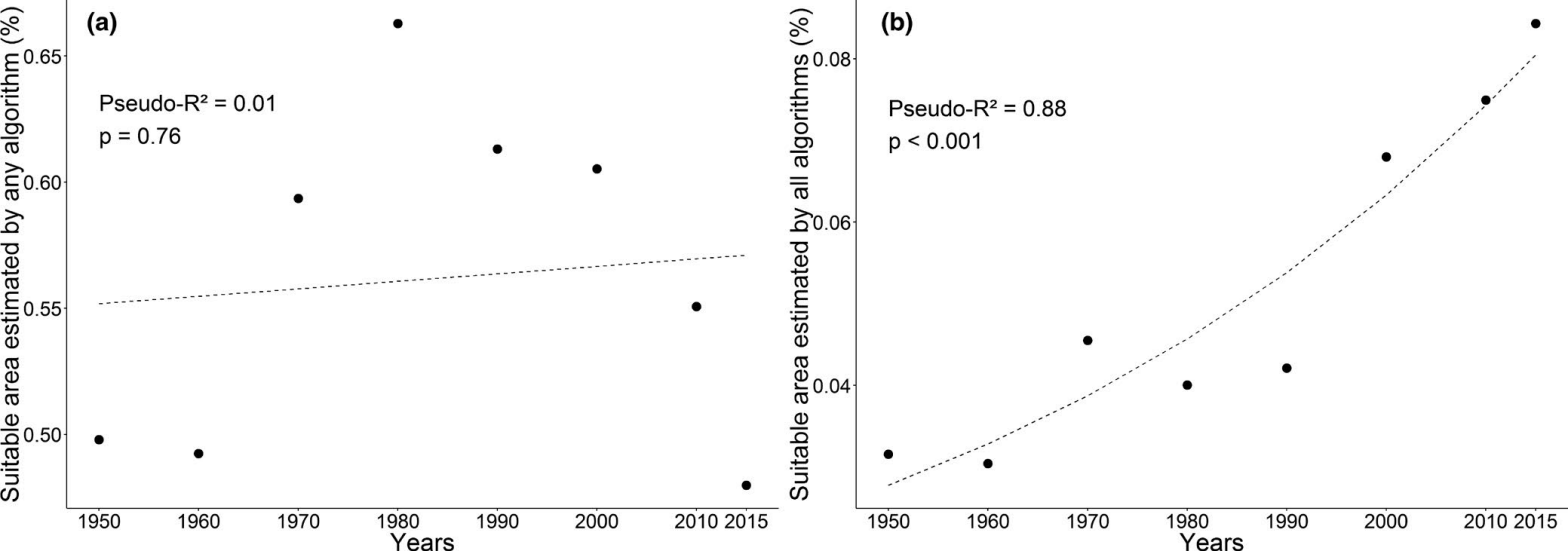
Investigating the decade effect hypothesis from two perspectives: (a) geographical distribution estimated by any algorithm (non‐conservatism approach); and (b) geographical distribution estimated by all algorithms (conservatism approach). In both approaches, we analyzed how newly added points over the decades alter the geographical distribution of *H*. *velatus*. Black spots represent the percentage of the predicted area by Ecological Niche Modeling for entire South America. The dashed line is the adjusted model estimated by the Beta regression of logit type

The modeling showed that the potential distribution of *H*. *velatus* (Figure 3) obtained from ENMs is highly variable over the decades with the addition of new records, in relation to its area of occurrence. In the period between 2000 and 2014, there was the greatest number of added points (Table 1), adding records of occurrence in Peru, Bolivia, Paraguay, Argentina, and mainly in Brazil, where it had about 91% of the total number of records. The increase in the sampling effort, mainly in Brazil, contributed to a better predictive adjustment of the distribution of occurrence of the species (Figures 1 and 3).

Over the decades, with the addition of new points, the potential geographical distribution of *H*. *velatus* reduced overprediction, the points adjusted better, giving better quality in predictive power. The results showed that the addition of the point recorded in 2015 in Goiânia caused an increase in the extension of the species potential distribution (Figure 1), and this increase was not predicted by three ENMs, predicted only in models 1 and 2, that is, areas of possible occurrence and tolerance. Even so, countries where the species has not yet been registered, such as Colombia, Ecuador, and Venezuela, appear as potential areas of occurrence. The greater the number of records (inventory execution), the accuracy of the distribution model increases (Hernandez et al., 2006; Pearson et al., 2007; Stockwell & Peterson, 2002). Accurate information on the distribution of species in countries with high biological diversity is scarce, and therefore, planning for conservation is done with low‐quality data (Lemes et al., 2011). Niche modeling is an important element in planning conservation and identifying areas where conservation efforts are most needed (Simião‐Ferreira & DeMarco, 2007). However, the difficulty of obtaining more restricted data, from environmental agencies, unpublished literature and without open access, is one of the problems for better accuracy and adjustment of models, showing the reality of studies on predictions.

*Histiotus velatus* is a species considered widely distributed, adapted to natural and urban environments, registered in Argentina, Bolivia, and Paraguay, while in Brazil it was registered in Mato Grosso, Piauí, Ceará, Distrito Federal, and the entire south and southeast region (Bianconi et al., 2007; Eisenberg & Redford, 1999; Emmons, 1997). Most of the studies that recorded the occurrence of *H*. *velatus* were based mainly on hammocks, and other methods were rarely used. This methodological bias raises doubts about the real distribution of this species, as it may be poorly sampled and its distribution may be even greater. Mist net is the main bat sampling technique (Kalko et al., 2007; Moras et al., 2013; Oprea et al., 2009; Stevens, 2013; Vieira et al., 2009) and is not efficient for species flying above the canopy, like many aerial insectivorous bats (Estrada‐Villegas et al., 2010; Kalko et al., 2007). The inclusion of other sampling methods (e.g., ultrasound detectors) may favor the increase in species registration (MacSwiney et al., 2008; Meyer et al., 2011; O’Farrell & Gannon, 1999).

The results showed that, in Brazil, the maps of potential distribution (Figure 1) show a trend of greater area of occurrence of the species understudy for the Southeast and South regions of Brazil. These regions are more anthropized, with few areas of native vegetation. In general, the collection of information on biodiversity tends to favor places with easy logistics, where there is already evidence of the occurrence of the species understudy (Hortal et al., 2008; Lobo, 2008). Updating the data depends on the researchers’ initiative (Amano et al., 2016; Girardello et al., 2018), and more populated places tend to have a greater sampling of biodiversity (Luck, 2007). This also makes research cheaper and more accessible to the researcher, especially when sampling is close to research centers (Hortal et al., 2007; Reddy & Dávalos, 2003; Romo et al., 2006).

In the last 25 years, there has been a significant increase in records of occurrences of species (Bernard et al., 2011), and this increase is probably due to new research centers located outside the South and Southeast regions. New research centers are important to reduce knowledge gaps and facilitate the understanding of the real distribution of Brazilian biodiversity. Even so, the North, Northeast, and Midwest regions were the areas with the smallest (Figure 1) points of occurrence. The North region presents a greater extension of forest areas, which aggregates Conservation Units and a greater concentration of indigenous lands, and the difficulty of access and the high logistical cost can be a limiting factor in data collection. The Northeast and Midwest regions, on the other hand, can be justified by the scarcity of sampling events, motivated by the lack of funding, time, and human resources (Beaman & Cellinese, 2012; Vollmar et al., 2010).

The results showed that there was no distance from the points of occurrence over the time of the research centers. The knowledge of the size of the species distribution, in some cases, may represent a sample bias limited by the geographic reach of the research centers (Hortal et al., 2007). The distance from the research centers may be the main factor that explains the sampling effort, which has given greater importance to the South and Southeast regions. The bias is a problem since priority areas for conservation are usually decided based on species richness (the fastest and cheapest way) in spite the are other methods (De Marco & Vianna, 2005). These vices have been known since the beginning of inventories (Hortal & Lobo, 2005) and are usually caused by the location of taxonomists, proximity to roads, proximity to cities (Beck & Kitching, 2007; Dennis & Thomas, 2000; Hortal et al., 2004), or the search for species of distribution already known. One way to alleviate one of these problems would be to support the establishment of researchers who work with bats in the priority regions mentioned in this work. Increasing the knowledge about the distribution of this (and other) species in South America.

Thus, overcoming the Wallacean gaps depends on investments in sampling efforts in places that are more distant from research centers and less accessible. In situ protection is the most viable and economical strategy (Loucks et al., 2008). Thus, by identifying potential areas of occurrence of species and, based on this, establishing priority areas for collections that aim to find new points of occurrence narrows the knowledge gap in the distribution of species. Still, the chances of success when planning biodiversity conservation and management are greater. Thus, based on the maps prepared, in the short term it will be possible to prioritize collection areas and plan field research to find new records more carefully. In the long run, the Wallacean deficit can be reduced in order to contribute to the preservation of *H*. *velatus* and the ecological processes in which it operates.

## CONFLICT OF INTEREST

There are no conflicts of interest.

## AUTHOR CONTRIBUTION

**Liriann Chrisley Nascimento Da Silva:** Writing‐review & editing (equal). **Rafaela Gonçalves Almeida:** Writing‐original draft (equal). **Pablo Henrique da Silva:** Writing‐review & editing (equal). **Monik Oprea:** Writing‐review & editing (equal). **Poliana Mendes:** Writing‐review & editing (equal). **Daniel Candido de Brito:** Writing‐review & editing (equal). **Thiago Bernardi Bernardi Vieira:** Formal analysis (equal); Supervision (lead).

## Data Availability

Data are available as supplementary material in Da Silva, Liriann Chrisley et al. (2021), temporal changes in the potential geographic distribution of *Histiotus velatus* (Chiroptera, Vespertilionidae), the “decade effect,” Dryad, Dataset, https://doi.org/10.5061/dryad.jsxksn097
